# Starch treatment improves the salivary proteome for subject identification purposes

**DOI:** 10.1007/s12024-023-00629-y

**Published:** 2023-04-21

**Authors:** Hannah Smith, Cecilia Giulivi

**Affiliations:** 1 Department of Molecular Biosciences, School of Veterinary Medicine, University of California Davis, Davis, CA USA; 2 MIND Institute, University of California at Davis Medical Center, Sacramento, CA USA

**Keywords:** Saliva, Proteome, Forensic identification, Starch, Method, Mass spectrometry

## Abstract

Identification of subjects, including perpetrators, is one of the most crucial goals of forensic science. Saliva is among the most common biological fluids found at crime scenes, containing identifiable components. DNA has been the most prominent identifier to date, but its analysis can be complex due to low DNA yields and issues preserving its integrity at the crime scene. Proteins are emerging as viable candidates for subject identification. Previous work has shown that the salivary proteome of the least-abundant proteins may be helpful for subject identification, but more optimized techniques are needed. Among them is removing the most abundant proteins, such as salivary α-amylase. Starch treatment of saliva samples elicited the removal of this enzyme and that of glycosylated, low-molecular-weight proteins, proteases, and immunoglobulins, resulting in a saliva proteome profile enriched with a subset of proteins, allowing a more reliable and nuanced subject identification.

**Supplementary Information:**

The online version contains supplementary material available at 10.1007/s12024-023-00629-y.

## Introduction

In forensic science, identifying bodies and perpetrators is crucial to solving crimes. Discriminating subjects associated with a crime scene as either non-perpetrators or perpetrators by using biological evidence is the most obvious. Still, few can help with timelines and gain more information than simple DNA genotyping.

Saliva can shed light on all these questions. Saliva—as a biological fluid—is already used in forensic analysis for various purposes since it is ubiquitously found at crime scenes (e.g., cigarette butts and envelopes or recovered from bite marks [1]) and easily obtainable through non-invasive procedures [2]. Salivary markers can help reconstruct events, such as identifying the type of food or drink the suspect had before or during a crime [3], detection of illegal substances [4], and microbiome profiling [5–7]. Markers in saliva can help with subject identification via salivary transcriptome [8], DNA [1, 9], and proteome [10–14].

Regarding subject identification using proteomes, proteins are usually used when DNA fingerprinting cannot be utilized because of low yield or high degradation. It has been reported that DNA has less stability and degrades faster than proteins based on chemical, biological, and environmental processes [15]. The salivary proteome has been used for subject identification by following two approaches: identifying genetically variant peptides [12–14] or utilizing those proteins with the highest variance across subjects [11]. In both cases, the interference of the most abundant proteins might obscure those less abundant, rare variants, undermining the identification of subjects.

**Fig. 1:**
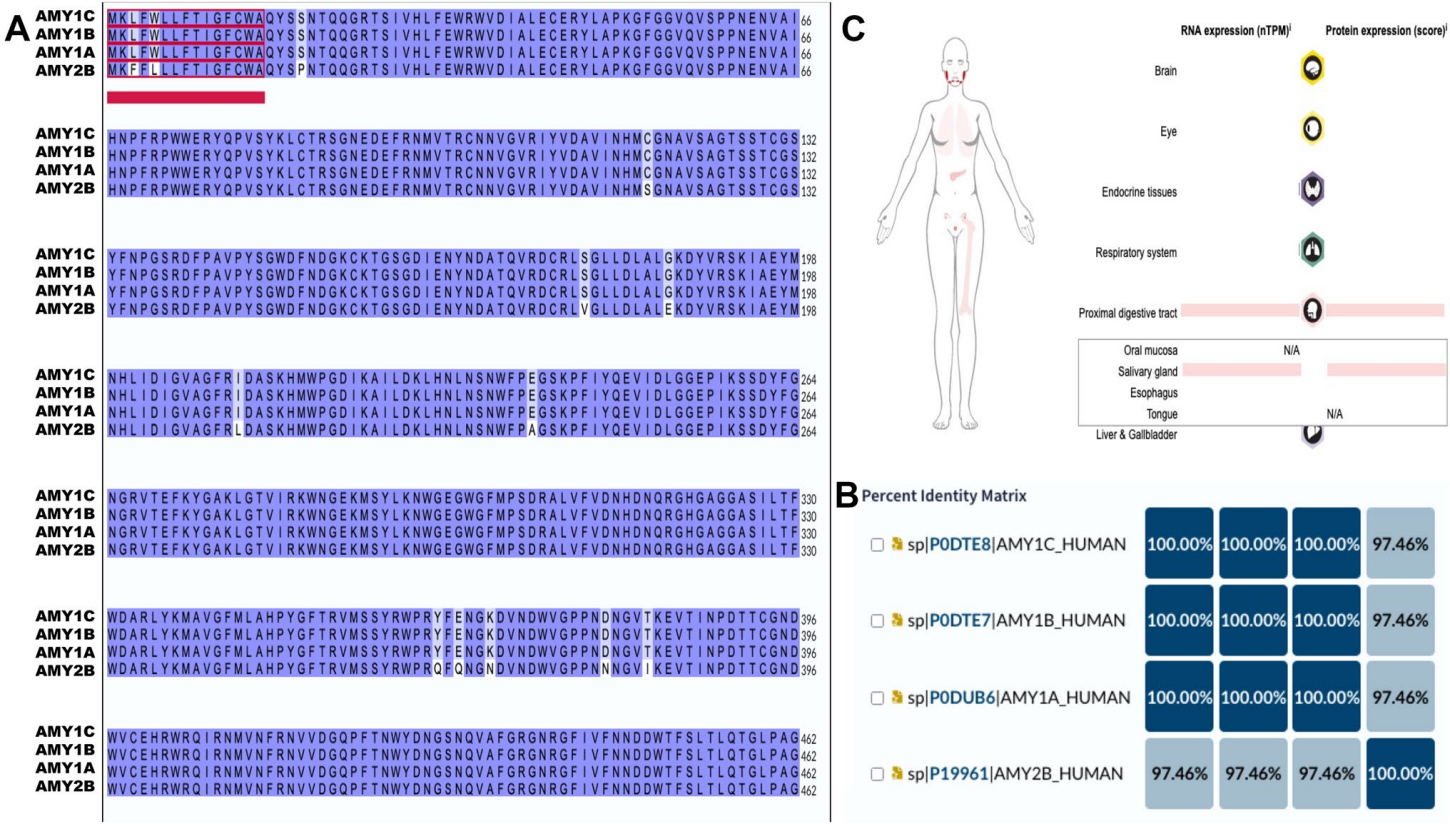
Alignment and expression of α-amylase isoforms in the body. **A** Alignment of primary sequences of isoforms AMY1A, AMY1B, AMY1C, and AMY2B. Alignment was performed with CLUSTAWL under Uniprot [31]. Similar amino acids are highlighted in blue, whereas the signal peptide is indicated in red. **B** Percentage of identity across isoforms. **C** Protein expression of AMY1A in the female body. Information retrieved from Human Protein Atlas [81]; www.proteinatlas.org). Image credit Human Protein Atlas (https://www.proteinatlas.org/ENSG00000237763-AMY1A/tissue)

Alpha amylase is among the most abundant proteins in saliva [16]. Amylases are secreted proteins that hydrolyze 1,4-alpha-glucoside bonds in oligosaccharides and polysaccharides and, thus, catalyze the first step in the digestion of dietary starch and glycogen [17]. The human genome encodes for a cluster of several amylases with high similarity (Fig. 1A, B), expressed mainly at high levels in either the salivary gland (AMY1A, AMY1B, AMY1C; Fig. 1C) or pancreas (AMY2A, AMY2B). Alternative splicing results in multiple transcript variants encoding the same protein. Salivary’s main proteins are amylases and other critical proteins such as histatins, statherins, cystatins, proline-rich proteins, mucins, and immunoglobulins [11, 18–25].

Based on the high abundance of amylase, we wanted to test the hypothesis of whether least-abundant proteins, identified by mass spectrometry and enhanced by the starch-mediated removal of amylase, were more suitable for subject identification. Given the numerous variables regarding the sources and composition of human saliva, many different approaches are required to compile a comprehensive catalog of the proteins that make up saliva with and without starch treatment. Mass spectrometry–based methods are of most significant utility because they are unbiased, requiring no prior knowledge of protein composition. In addition, once proteins are identified through protein databases, insights into protein function, localization, and cofactor requirement, among others, are very relevant and complementary to mass spectrometry methods. This approach, coupled with tandem MS-based strategies, in combination with liquid chromatography and electrospray ionization, has been used with database search algorithms to identify proteins [26, 27]. These so-called bottom-up methods have been used for various studies of biological fluids, including saliva [28].

## Materials and methods

### Sample collection

Fifteen 2.0-ml unstimulated saliva samples were taken from female volunteers aged 21–61 years. The only identifying information received from each subject was age and sex. The collection of samples occurred on a single day (February 4, 2022) from 11:00 a.m. to 2:00 p.m. Subjects had not eaten, drunk, or performed oral hygiene routines for 30 min before providing samples. Using a sterile Corning tube given by the researcher, subjects provided saliva after tilting their heads back and letting it pool for 60 s. Samples were collected according to informed consent policies by the institutional review board.

### Saliva preparation

Two aliquots (1 mL) from each of the saliva samples were treated with (*n* = 15) or without (*n* = 15) starch to remove amylase by following the method described by [29] with modifications. The starch used in this study was from potatoes because, compared to other starches, it has a low lipid level (Sigma-Aldrich, S2004). A starch solution was prepared (20 g/l in double-distilled Milli-Q water). It was cleaned of spurious material by washing it 3 times in cold double-distilled Milli-Q water and centrifuged at 5000 rpm for 5 min. Each microcentrifuge tube contained 1 ml of saliva and either 1 ml of the clean and hydrated starch solution (treated) or 1 ml of water (non-treated). The tubes were stirred for 5 min and then centrifuged at 16,000 × g for 10 min at 4 °C. All samples were concentrated and partly delipidated by acetone precipitation by adding four volumes of − 20 °C acetone (analytical grade; Sigma-Aldrich, St Louis) to each sample. Acetone-containing mixtures were vortexed and placed at − 20 °C for 24 h. Samples were centrifuged at 16,000 × g for 10 min at 4 °C. After pouring off the supernatant, the pellets were resuspended and washed twice with − 20 °C acetone, spinning each wash at 16,000 × g for 10 min at 4 °C. After removing the supernatant from the final wash, the samples were placed in the SpeedVac for 15 min to remove residual acetone.

### Proteomics

All samples (treated and non-treated with starch) were analyzed using liquid chromatography coupled to tandem mass spectrometry for the protein profiles of the saliva, following published methods [11]. All chemicals utilized in this study were of analytical grade or higher. The protein pellet was solubilized in 100 µl of 6-M urea/50-mM ammonium bicarbonate, pH 8. Then, 2.5 µl of 5 mM dithiothreitol (DTT) was added and incubated for 30 min at 37 °C. Twenty microliters of 5 mM iodoacetamide (IAA) was added and set for 30 min at room temperature in the dark. Twenty *µ*l of DTT was added to quench IAA and incubated for 10 min at room temperature. Porcine Lys-C/trypsin mix, mass spectrometry grade (Promega Corporation), was added in a 1:25 ratio and set for 4 h at 37 °C. Six hundred microliters of 50 mM ammonium bicarbonate was added to dilute the urea concentration to < 1 M and incubated overnight at 37 °C. The next day, the digest was desalted with a Macro Spin Column (The Nest Group, Inc.). Depending on the sample amount, 10–100 μg of a digest prepared from each sample was analyzed by mass spectrometry.

Digested peptides were analyzed by LC–MS/MS on a Thermo Scientific Q Exactive Plus Orbitrap mass spectrometer in conjunction with Proxeon Easy-nLC II HPLC (Thermo Scientific) and Proxeon nanospray source. The digested peptides were loaded onto a 100 µm × 25 mm Magic C18 100 Å 5U reverse-phase trap where they were desalted online before being separated using a 75 µm × 150 mm Magic C18 200 Å 3U reverse-phase column. Peptides were eluted using a 140-min gradient with a flow rate of 300 nl × min^−1^. A MS survey scan was obtained for the *m*/*z* range 350–1600, and MS/MS spectra were acquired using a top 15 method, where the top 15 ions in the MS spectra were subjected to high energy collisional dissociation. An isolation mass window of 1.6 m/*z* was for the precursor ion selection, and a normalized collision energy of 27% was used for fragmentation. A 15-s duration was used for the dynamic exclusion.

Database searching–tandem mass spectra were extracted by Proteome Discoverer v.2.2. Charge state deconvolution and deisotoping were not performed. All MS/MS samples were analyzed using X! Tandem (The GPM, thegpm.org; version X! Tandem Alanine (2017.2.1.4)). X! Tandem was set up to search the UniProt Human proteome database plus 110 common laboratory contaminants and an equal number of decoy sequences (147,936 entries total), assuming the digestion enzyme trypsin. X! Tandem was searched with a fragment ion mass tolerance of 20 ppm and a parent ion tolerance of 20 ppm. Glu- > pyro-Glu of the *N*-terminus, ammonia-loss of the *N*-terminus, Gln- > pyro-Glu of the *N*-terminus, deamidation of Asn and Gln, oxidation of Met and Trp, and dioxidation of Met and Trp were specified in X! Tandem as variable modifications.

Scaffold (v.Scaffold_4.9.0, Proteome Software) was used to validate MS/MS-based peptide and protein identifications. Peptide identifications were accepted if they could be established at greater than 98.0% probability by the Scaffold Local false discovery rate (FDR) algorithm. Peptide identifications were also required to exceed specific database search engine thresholds. X! Tandem identifications required at least − log(*E*-value) of 2. Protein identifications were accepted if they could be established at greater than 5.0% probability to achieve an FDR less than 5.0% and contained at least 1 identified peptide. This resulted in a peptide decoy FDR of 0.7% and a protein decoy FDR of 0.66%. Proteins that contained similar peptides and could not be differentiated based on MS/MS analysis alone were grouped to satisfy the principles of parsimony. Proteins sharing significant peptide evidence were grouped into clusters.

### Statistical analysis

All analyses (indicated in the text) were performed with GraphPad using either a paired Student *t*-test or a chi-square test (proportions). Data were analyzed using principal component analysis performed with ClustVis 2.0 [30].

## Results

### Analysis of the salivary proteome

**Fig. 2:**
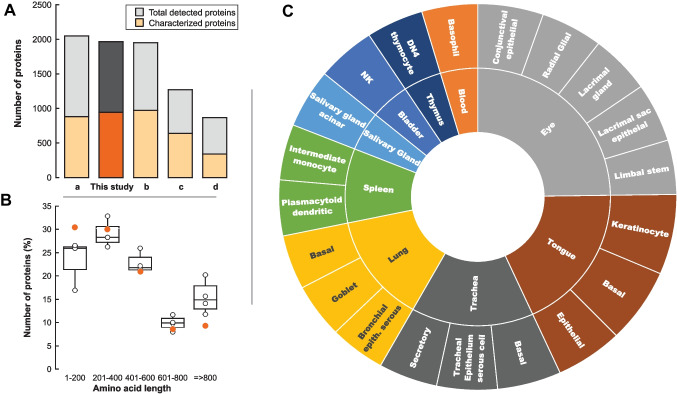
Characterization of the salivary proteome from 15 females. **A** The total number of proteins detected in each of the studies (grey) and that of the ones characterized, reviewed, and present in the UniProt database (orange). These numbers were calculated from the following studies: **a** parotid and submandibular/sublingual saliva [34]; **b** whole saliva from females [11]; **c** whole saliva [33]; **d** whole saliva from females aged 20–30 years and 55–65 years [32]. **B** Distribution of proteins based on their amino acid length. The amino acid lengths of all reviewed and characterized proteins from each of the studies—including the current one—were obtained from UniProt. The results were plotted using a box plot. Each box encloses 50% of the data, with the median value of the four studies displayed as a line. The top and bottom of the box mark the limits of ± 25% of the variable population. The lines extending from the top and bottom of each box mark the minimum and maximum values within the data set that fall within an acceptable range. Any value outside this range, called an outlier, is displayed as an individual point. Data from the current study are shown in orange. **C** Tissue and cell distribution of the salivary proteome from this study. Reviewed and characterized proteins were analyzed using EnrichR [82] and the Human Proteome Map [83]. Only the top 20 data are shown based on the negative log of q-value. The q-value is an adjusted *p*-value calculated using the Benjamini–Hochberg method for correction for multiple hypotheses testing. Data were obtained for tissues (inner doughnut) and cell type (outer doughnut)

Untargeted proteomics of saliva samples from 15 females aged 21 to 61 detected 1017 proteins, from which 945 were present in the protein Uniprot database [31]. The total number of proteins and the reviewed and characterized ones were within the values previously reported by other studies (Fig. 2A; Supplementary file [11, 32–34]). The salivary proteome was within a wide molecular range, with those with 400 or fewer amino acids comprising approximately 65% of total proteins (Fig. 2B), like those of the four studies mentioned above (Fig. 2B) and consistent with others [35–40]. In this study, the most abundant proteins with 400 or fewer amino acids were consistent with approximately identical masses of salivary peptides known as cystatins (*n* = 7, 98 to 146 amino acids), proline-rich proteins (*n* = 13, 89 to 310 amino acids), defensins (*n* = 1, 94 amino acids), and calgranulins (*n* = 3, 92 to 114 amino acids). Other protein-detected proteins relevant to saliva were immunoglobulins, keratins, fatty acid-binding proteins, histatins, kallikrein, lysozyme, proline-rich proteins, and superoxide dismutase, which also have been identified by other proteomic techniques [41–49].

More than 1000 different proteins have already been identified in saliva, from which only a fraction (20 to 30%) is directly derived from salivary glands’ secretion [34, 50], suggesting a significant contribution of other sources, namely plasma/serum, gingival crevicular fluid, minor glands, and oral epithelia [44, 48, 51–53]. Consistent with these reports, our study identified proteins from the oral cavity (tongue, salivary glands; Fig. 2C), airway (trachea, lung), blood (spleen, blood), and similar to the tear fluid proteome, which is produced by the lacrimal gland (eye; Fig. 2C) as reported before [34].

### Effect of starch treatment on the salivary proteome

**Fig. 3:**
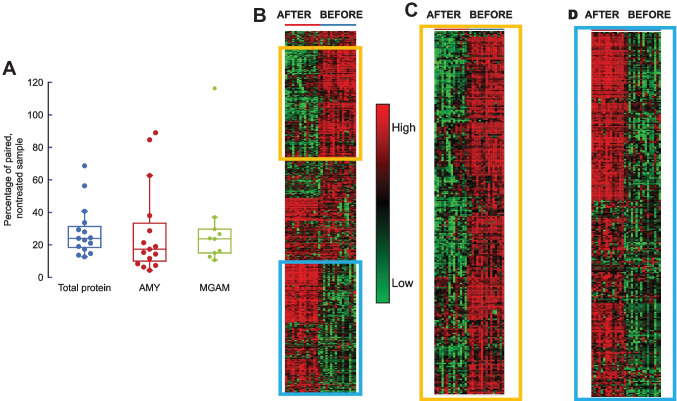
Effect of starch treatment on the salivary proteome of 15 females. **A** Abundance of total proteins/subject with and without starch treatment (blue), the abundance of amylase/subject with and without starch treatment (red), and of MGAM (green). **B** Heat map visualizing the protein abundance distribution across all samples. Subjects on *X*-axis, proteins on *Y*-axis. Proteins depleted (**C**) or enriched (**D**) by the starch treatment

Amylase abundance in untreated samples was within reported values (mean ± SD, *n* = 15, = 17 ± 11% of total proteins; 12 to 30% [11, 54]). As expected, the intensity of enzymes for which starch is a substrate, such as amylase (AMY) and maltase-glucoamylase (MGAM), was decreased to 29 ± 28% (*p* = 0.001) and 28 ± 30% (*p* = 0.016) of their respective original contents in untreated samples (Fig. 3A). Unexpectedly, the starch treatment decreased the total abundance of proteins to 28 ± 16% (paired Student’s test *p* = 3.5 × 10^−5^) of those without starch, suggesting unspecific protein adsorption to starch other than amylase (Fig. 3A). However, a more detailed analysis revealed that starch treatment unexpectedly affected the salivary proteome.

**Fig. 4:**
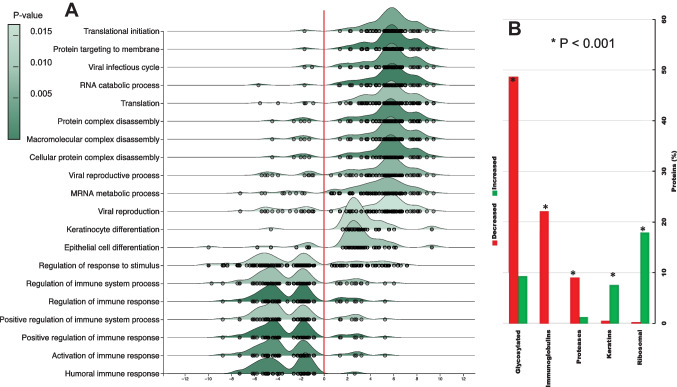
Biological function and protein features associated with the most discriminating proteins before and after starch treatment. **A** Statistically significant proteins between treatments (*n* = 601) were obtained through a pair-wise test using Limma and selected by the adjusted *p*-value set at 0.05 or below. This analysis revealed 601 different proteins. These were used as input for the ridgeline diagram of enriched functions. All analysis was performed with NetworkAnalyst [55]. Only the top 20 are shown. Results were organized by log2 fold ratio (*X*-axis), and the shade of green indicates the significance. **B** The statistically different proteins between treatments were analyzed for glycosylation and if they belong to the following families: immunoglobulins, proteases or peptidases, keratins, and ribosomal. Analysis was performed with Uniprot. * indicates the *p*-value of the Chi-squared test for each outcome between treatments

From the 945 salivary proteins from 15 females before and after starch treatment, 826 were matched to a single gene, and 8 were unmatched; all 834 were kept for further analysis. Statistical analyses (Limma) between the two paired groups (before and after starch treatment) normalized to their sum and adjusted by the variance-stabilizing normalization followed by quantile normalization (analysis performed using NetworkAnalyst version 3. [55]) indicated that samples after starch treatment were depleted in 309 (Fig. 3B, C, orange box) and enriched in 292 proteins (Fig. 3B, D, blue box; Supplementary file). Notably, those depleted by the starch treatment were associated with immunity and proteolysis, and they were mainly glycosylated (Fig. 4A; Supplementary file). In contrast, those 292 proteins enriched by starch treatment were associated primarily with translation (the ribosomal family) and cytoskeleton (keratins; Fig. 4B; Supplementary file).

Thus, the adsorption of proteins to starch was not unspecific but targeted not only its natural enzyme amylase but also glycosylated proteins. This result is supported by the binding of the active site of amylase to saccharide hydrolysate of starch, and amylase also has several oligosaccharide-binding sites, which enhance the affinity of α-amylase to the starch granules [56].

### Value of starch treatment for subject identification based on salivary proteome

Given that many proteins were depleted after starch treatment, including immunoglobulins, glycosylated proteins, and proteases, we wanted to assess whether the remaining proteins had more discriminatory power to identify subjects.

**Fig. 5:**
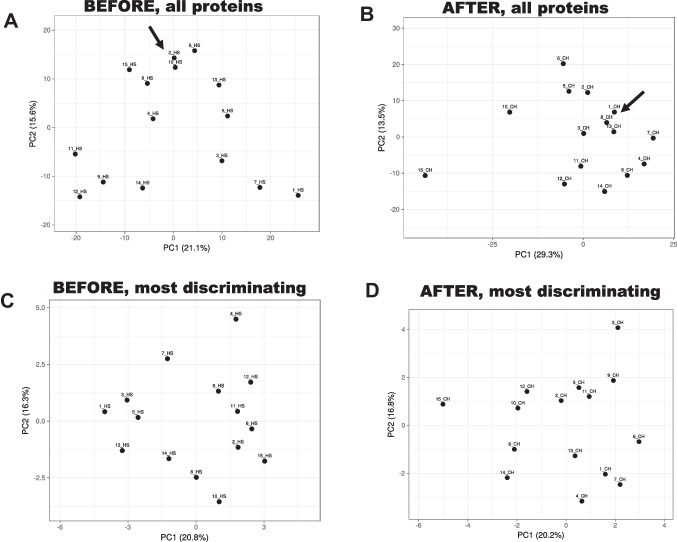
Principal component analysis of the salivary proteome under different conditions. PCA (performed with ClustVis 2.0 [30]) was applied by utilizing proteins (normalized by variance-stabilizing normalization followed by quantile normalization) for each treatment. Unit variance scaling was applied to rows; singular value decomposition with imputation was used to calculate principal component analysis (to all panels). Other options were set as follows: no data transformation and no collapse of columns with similar annotations were performed; the maximum percentage of unavailable data allowed in both rows and columns was set at 99.99; row centering; no removal of constant columns; row scaling was based on unit variance scaling, and the PCA method was calculated by using singular value decomposition. Panels A and B were obtained by running a PCA with all proteins before (**A**) and after (**B**) starch treatment. Lower panels were obtained with the most discriminating proteins before (**C**) and after (**D**) starch treatment

To this end, we used principal component analysis to analyze the data because it allows for analyzing large datasets with many parameters per subject by reducing the dataset complexity to increase the interpretability of data while preserving the complete information. The data are linearly transformed into a new coordinate system where most of the data variation is observed with fewer dimensions than the original data (usually in a 2-dimensional plot) to visually identify clusters of closely related data points. Principal component analyses (PCAs) were created for each set of normalized samples before and after starch treatment (Fig. 5AB). Visual inspection of the PCA plots before and after starch treatment indicated a broad sample distribution under both conditions with similar contributions of principal components 1 and 2 (29.3 vs. 21.1% for PC1 and 13.5% vs. 15.6% for PC2; Fig. 5AB), but under each condition, few samples appeared clustered (Fig. 5A: 2_HS and 10_HS; Fig. 5B: 1_CH, 8_CH, and 13_CH; arrows). Selecting the top 3 proteins from each principal component whose sum accounts for 80% of the variability (27 proteins from 9 principal components and 24 from 8 principal components for before and after starch treatment, respectively; Supplementary file; Table 1) resulted in a more suitable separation of samples (Fig. 5C, D). Notably, no protein duplicates were found before and after starch treatment, and 9 were among 94 proteins previously reported as discriminating ones utilizing a different protocol of normalization and identification [11].

**Table 1:**
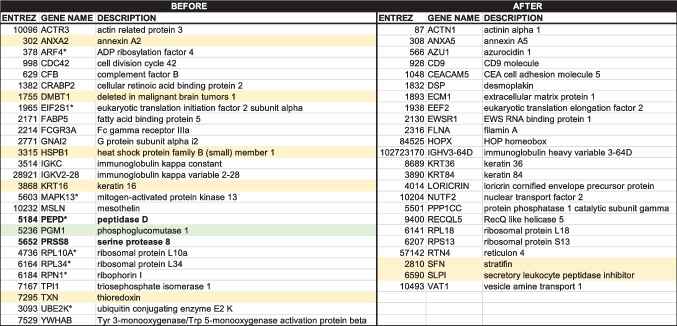
Top discriminating proteins across subjects before and after starch treatment

The PCA analysis (Fig. 5C, D) obtained the top discriminating proteins with their Entrez GI number, gene name, and description. In orange, previously identified proteins with discriminating power [11]. In green, shown is the only age-dependent protein. *Proteins not present in 80% or more of samples. In bold, proteases

While the two PCA plots obtained with the most discriminating proteins before and after starch treatment seemed comparable, we observed two clear advantages of using the starch treatment: (i) from the selected 27 discriminating proteins obtained before starch treatment, 8 were not detectable in 80% or more of the samples; however, they were still included in the analysis as “filtered values” were replaced by 1/5 of the lowest positive value reported for the particular feature [57]. However, none of the 24 discriminating proteins obtained after starch treatment were missing in 80% or more of the samples, overcoming issues with putative data bias. (ii) Regarding protein stability during processing and storage, the discriminating proteins obtained before starch treatment included two proteases, whereas none were among the 24 obtained after starch treatment.

Finally, the discriminating power of these 51 proteins was not associated with age, as only one showed an age dependency, phosphoglucomutase 1 (Table 1 in green; PGM1 vs. age; *r* (14) = 0.786; *P* = 0.0005) consistent with other studies showing a statistically negative correlation between age and PGM activity in the aortic, pulmonary artery, and coronary artery tissue [58] and red blood cells [59].

## Discussion

Analysis of human body fluid proteome has become one of the most promising approaches to discovering biomarkers of diagnosis and prognosis of diseases. This analysis is challenging because body fluids have a complex protein composition. In addition, several variables must be considered, including sample preparation and handling, protein prefractionation, depletion of highly abundant proteins, quantification of proteins, and data analysis. Understanding the inherent limitations of these steps is critical to design the best approach and analysis of a specific body fluid proteome. In this study, the body fluid used was human saliva, which is constituted by the secretion of multiple salivary glands (parotid, sublingual, and submandibular), other minor glands located underneath the oral mucosa, as well as proteins from plasma/serum, gingival crevicular fluid, and oral epithelia [44, 48, 51–53]. As the most abundant proteins in saliva are amylases, cystatins, and immunoglobulins [43, 60], we reasoned that depletion of, for instance, amylase, before mass spectrometry analysis would improve mapping and identification of salivary proteins [61], resulting in a suitable salivary proteome for subject identification.

Our study showed that starch treatment of saliva samples significantly depleted amylase (as expected) and other proteins, primarily glycosylated and low molecular weight. However, the decrease of ~ 70% protein content did not affect subjects’ separation based on their salivary proteome. Indeed, PCA plots showed almost similar discriminating power across samples before and after starch treatment (Fig. 5C, D), requiring 27 and 24 proteins, respectively. In both cases, using 24 or 27 proteins from the original 945 ones for subject identification is a significant improvement. Regarding salivary proteins that change with age, as our cohort of subjects had a broad range of age (from 21 to 61 years), we explored whether any of these proteins showed an age dependency. Only PGM1 showed an age dependency, indicating that differences across subjects were not based on age, consistent with another study with similar subject identification purposes [11].

Interestingly, a study found significant age-related differences in gene expression in the human female parotid gland [62]. Still, few and often conflicting age-related differences have been reported at the proteomic level. Some studies have reported no significant changes with age in saliva composition [63], salivary flow, and buffering capacity [64] of saliva. In contrast, mucins decreased [65, 66] while transforming growth factor-α, IgG, and IgA increased with age [67, 68] in whole saliva. No age-associated changes in parotid saliva were observed for total protein content, amylase, lactoferrin, secretory IgA, and proline-rich proteins [69–71]. However, other studies reported increased total protein, secretory IgA, and lactoferrin [70, 72] and reduced amylase activity with age in parotid saliva [70].

Starch treatment had two significant benefits when focusing on the discriminating proteins: (i) all were present in 80% or more of the samples, and (ii) none were proteases. Detecting all discriminatory proteins in most samples overcomes issues associated with mathematically replacing missing values. For the second point, it has been reported that amylase removal increases the stability of other salivary proteins when stored at room temperature [73]. While our study did not test whether starch treatment increased protein stability, the treatment did deplete the samples of several proteases and peptidases (Table 1), suggesting that by lowering the proteases’ content, the stability of remaining proteins is enhanced. In addition, the step utilizing acetone to precipitate and concentrate salivary proteins enhances the denaturation of proteins and delipidation, and, very likely, stability [as another study reported by using ethanol [73] as remaining proteases and peptidases are denatured by this organic solvent.

One clear advantage of using salivary proteome over DNA is when there is significant DNA degradation or low yield, both of which undermine suitable DNA profiling. A clear example is a study reporting significant amylase activity in saliva samples left on 26-year-old envelopes stored at room temperature [74]. Moreover, recalculating data from that study, amylase protein recovery from chewing gum, envelopes, and cigarette butts was ~ 6-fold that of DNA (5.73 ± 0.57 ng amylase protein/ng DNA; *n* = 32; *p* = 0.0009). Since amylase protein content is ~ 60% of total salivary proteins [29, 46] and 40 to 80% calculated from total salivary protein content = 0.25 to 1 mg/ml [75] and amylase concentration = 100–800 µg amylase/ml saliva [76], indicating that, even for the lowest amylase content of the 26-year-old samples (0.3 ng), a 0.6 ng protein would be above the typical mass spectrometer detection limit (~ 500 fg for 50 kD protein [77]) without the need of amplifying the template as it is required when using DNA.

Future studies should address its value compared to that of DNA profiling, explore the stability of salivary proteins with exposure to sunlight or blue light (450 nm) or the reagent on Phadebas paper, both direct methods used to locate saliva stains in crime scenes [78], various storage conditions and protein extraction from different materials (e.g., cigarette butts, chewing gum, envelopes, stamps on letters, glasses, silverware, among others), and larger cohorts. Currently, it is unknown whether the most discriminating proteins used for subject identification are constantly changing as a result of minor physiological or pathological events in a person or if they are highly stable even in the face of major biological events [61]; however, several studies showed no differences in the proteomes of dry or wet saliva samples [79, 80].

Finally, starch treatment resulted in the depletion of amylase and other glycosylated proteins, resulting in a salivary proteome that allows the discrimination of subjects.

## Key points

1. Saliva is a common biological fluid found at crime scenes and contains identifiable components.
2. DNA has been the most prominent identifier, but analyzing it can be complex due to low yields and issues with preservation at the crime scene.
3. Salivary proteome obtained by mass spectrometry is a sensitive, non-biased, and useful tool to identify subjects.
4. Proteins, particularly the least-abundant ones in the salivary proteome, are emerging as viable candidates for subject identification.
5. Removing the most abundant proteins through starch treatment can enrich the proteome profile and lead to more reliable and nuanced subject identification.

### Supplementary Information

Below is the link to the electronic supplementary material.

Supplementary file1 (XLSX 1337 KB)

## Data Availability

The dataset used and analyzed during the current study was included as Supplementary Information.
